# *Mangifera indica* L. Leaf Extract in Combination With Luteolin or Quercetin Enhances VO_2_peak and Peak Power Output, and Preserves Skeletal Muscle Function During Ischemia-Reperfusion in Humans

**DOI:** 10.3389/fphys.2018.00740

**Published:** 2018-06-08

**Authors:** Miriam Gelabert-Rebato, Julia C. Wiebe, Marcos Martin-Rincon, Nigel Gericke, Mario Perez-Valera, David Curtelin, Victor Galvan-Alvarez, Laura Lopez-Rios, David Morales-Alamo, Jose A. L. Calbet

**Affiliations:** ^1^Department of Physical Education and Research Institute of Biomedical and Health Sciences (IUIBS), University of Las Palmas de Gran Canaria, Las Palmas de Gran Canaria, Spain; ^2^Nektium Pharma, Las Palmas de Gran Canaria, Spain

**Keywords:** sprint exercise, polyphenols, antioxidants, fatigue, recovery

## Abstract

It remains unknown whether polyphenols such as luteolin (Lut), mangiferin and quercetin (Q) have ergogenic effects during repeated all-out prolonged sprints. Here we tested the effect of *Mangifera indica* L. leaf extract (MLE) rich in mangiferin (Zynamite®) administered with either quercetin (Q) and tiger nut extract (TNE), or with luteolin (Lut) on sprint performance and recovery from ischemia-reperfusion. Thirty young volunteers were randomly assigned to three treatments 48 h before exercise. Treatment A: placebo (500 mg of maltodextrin/day); B: 140 mg of MLE (60% mangiferin) and 50 mg of Lut/day; and C: 140 mg of MLE, 600 mg of Q and 350 mg of TNE/day. After warm-up, subjects performed two 30 s Wingate tests and a 60 s all-out sprint interspaced by 4 min recovery periods. At the end of the 60 s sprint the circulation of both legs was instantaneously occluded for 20 s. Then, the circulation was re-opened and a 15 s sprint performed, followed by 10 s recovery with open circulation, and another 15 s final sprint. MLE supplements enhanced peak (Wpeak) and mean (Wmean) power output by 5.0–7.0% (*P* < 0.01). After ischemia, MLE+Q+TNE increased Wpeak by 19.4 and 10.2% compared with the placebo (*P* < 0.001) and MLE+Lut (*P* < 0.05), respectively. MLE+Q+TNE increased Wmean post-ischemia by 11.2 and 6.7% compared with the placebo (*P* < 0.001) and MLE+Lut (*P* = 0.012). Mean VO_2_ during the sprints was unchanged, suggesting increased efficiency or recruitment of the anaerobic capacity after MLE ingestion. In women, peak VO_2_ during the repeated sprints was 5.8% greater after the administration of MLE, coinciding with better brain oxygenation. MLE attenuated the metaboreflex hyperpneic response post-ischemia, may have improved O_2_ extraction by the *Vastus Lateralis* (MLE+Q+TNE vs. placebo, *P* = 0.056), and reduced pain during ischemia (*P* = 0.068). Blood lactate, acid-base balance, and plasma electrolytes responses were not altered by the supplements. In conclusion, a MLE extract rich in mangiferin combined with either quercetin and tiger nut extract or luteolin exerts a remarkable ergogenic effect, increasing muscle power in fatigued subjects and enhancing peak VO_2_ and brain oxygenation in women during prolonged sprinting. Importantly, the combination of MLE+Q+TNE improves skeletal muscle contractile function during ischemia/reperfusion.

## Introduction

Fatigue is a complex process which may originate in any structure intervening in the production and control of muscle contractions. Performance-enhancing compounds may exert their effects by facilitating energy supply and utilization, easing central command and motor control and reducing the negative effects caused by energy depletion, shortage of O_2_, metabolite accumulation, and reactive oxygen and nitrogen species (RONS) on force generation, muscle contraction activation and afferent feedback. Among the natural substances that may have performance-enhancing properties (ergogenic effects) several polyphenols have been investigated (González-Gallego et al., 2010; Braakhuis and Hopkins, 2015). Most polyphenols may act as antioxidants (Sandoval-Acuña et al., 2014), signaling molecules, or hold anti-inflammatory (Luczkiewicz et al., 2014), anti-aging (Khurana et al., 2013; Menendez et al., 2013), neuromodulatory and neuroprotective (Campos-Esparza et al., 2009; Luo et al., 2017) properties, which may confer their ergogenic potential. Most of these effects have been demonstrated in cell culture or animal models, in many instances using supra-physiological doses (González-Gallego et al., 2010). Moreover, studies performed in humans have reported divergent results which have been attributed to differences in the exercise model, fitness level of the study population and the type of polyphenol tested (González-Gallego et al., 2010). The ergogenic potential of some polyphenols such as luteolin and mangiferin remains unknown, and the effects of quercetin on performance during repeated all-out prolonged sprints is yet to be studied in humans.

Mangiferin (2-b-D-glucopyranosyl-1,3,6,7-tetrahydroxy-xanthone) is a xanthone (non-flavonoid polyphenol) originally isolated from *Mangifera indica* L. *(Anacardiaceae)*, present in abundance in mango leaves and other plants (Masibo and He, 2008). Mangiferin is considered a “super antioxidant” capable of specifically protecting against free radical production by the Fenton reaction due to its iron-chelating properties. The Fenton reaction is thought to play an important role as a source of RONS during sprint exercise (Morales-Alamo and Calbet, 2014), due to the marked acidification elicited by the high glycolytic rates attained during this type of exercise. Acidosis accelerates hydroxyl radical production by the Fenton reaction and reduces the activities of the antioxidant enzymes glutathione peroxidase, glutathione S-transferase, and glutathione reductase (Ying et al., 1999).

Mangiferin has powerful free radical scavenging properties and has been shown to attenuate ischemia/reperfusion injuries in diabetic rats (Suchal et al., 2017), but it remains unknown whether mangiferin attenuates the effects of ischemia/reperfusion in humans. Mangiferin can traverse the blood-brain barrier and modulate neurotransmission, K^+^ channels and nociception (Rauf et al., 2017). Strong stimulation of type III and IV afferents by metabolite accumulation during sprint exercise (Cheetham et al., 1986; Morales-Alamo et al., 2012), particularly H^+^ and lactate (Light et al., 2008) are likely involved in the perception of effort and exercise-induced pain. III/IV muscle afferents discharge inhibits corticospinal drive and could contribute to limit exercise capacity or enhance fatigue sensation (Amann and Dempsey, 2008; Rossman et al., 2012; Sidhu et al., 2014; Kennedy et al., 2015). Mangiferin has properties which may attenuate III/IV muscle afferent discharge during exercise, either by reducing RONS-mediated stimulation of III/IV muscle afferents or by downregulating the glycolytic rate and interstitial K^+^ accumulation.

Quercetin is a well-studied flavonoid polyphenol which may improve performance during prolonged exercise (Kressler et al., 2011; Myburgh, 2014), although its effects in athletes are unclear (Braakhuis and Hopkins, 2015). Quercetin is found in several fruit and vegetables, including mangoes. Although quercetin has a low bioavailability due to its poor intestinal absorption (Graefe et al., 2001), this may be improved by an oleaginous vehicle (Tran et al., 2014) such as tiger nut extract. In animal models, quercetin attenuates ischemia/reperfusion injuries in several tissues (Shoskes, 1998; Cho et al., 2006; Annapurna et al., 2009) including skeletal muscle (Ekinci Akdemir et al., 2016). A potential sex dimorphism in the responses to polyphenol supplementation have not been specifically tested, although quercetin (like mangiferin) is a phytoestrogen, capable of binding to and activating estrogen receptors (Wilkinson et al., 2015).

Luteolin (30, 40, 50, 70-tetrahydroxyflavone) is one of the most abundant flavones and, like mangiferin and quercetin, is a potent antioxidant and inhibitor of xanthine oxidase (XO) (Nagao et al., 1999; Pinto et al., 2005; Paredes-Gonzalez et al., 2015; Niu et al., 2016; Nile et al., 2017). Luteolin is also a NADPH oxidase (nicotinamide adenine dinucleotide phosphate-oxidase; NOX) inhibitor (Makino et al., 2013; Xia F. et al., 2014). Both enzyme activities, XO and NOX, play a critical role in RONS generation during intense exercise (Morales-Alamo and Calbet, 2014) and ischemia/reperfusion events (Berry and Hare, 2004). Luteolin mitigates ischemia/reperfusion damage in cell cultures (Tian et al., 2018) and animals (Karakaş et al., 2014; Hong et al., 2017; Liu et al., 2017; Luo et al., 2017; Du et al., 2018).

Flavonoids may facilitate an increase in mitochondrial Ca^2+^ concentration by acting on the mitochondrial Ca^2+^ uniporter (Montero et al., 2004). This may up-regulate the respiratory rate and ATP production and stimulate endothelial nitric oxide synthase (eNOS), increasing nitric oxide (NO) production (Duarte et al., 2014; Si et al., 2014; Cheng et al., 2018). The vasodilation induced by NO may enhance oxygen delivery to the active muscles also helping to improve performance (Calbet and Lundby, 2012; Si et al., 2014). Although it has been reported that the effects on performance could be enhanced when flavonoids are given in combination (MacRae and Mefferd, 2006), few studies have examined the effects of flavonoid combinations on exercise performance (MacRae and Mefferd, 2006; Deley et al., 2017; Overdevest et al., 2018).

Interestingly, the cytoprotective effects of flavonoids against ischemia/reperfusion may be enhanced by exercise (Chang et al., 2014). However, no single study to date has determined the efficacy of natural polyphenols in mitigating the deterioration of skeletal muscle contractile function after short ischemia/reperfusion in humans. Ischemia/reperfusion is a phenomenon that may occur during isometric contractions (Wigmore et al., 2006; Thompson et al., 2007). Tempol, an antioxidant mimicking superoxide dismutase, preserves skeletal muscle mitochondrial respiratory function during reperfusion in rodents (Charles et al., 2017). Likewise, quercetin has been shown to protect skeletal muscle from ischemia/reperfusion injury in rats (Ekinci Akdemir et al., 2016). Thus, for this study we hypothesized that natural polyphenols might also facilitate the recovery of exercise performance after ischemia/reperfusion in humans by easing mitochondrial O_2_ utilization after ischemia.

Therefore, the main aim of this study was to test whether a mango leaf extract (MLE) (60% weight mangiferin) administered in two different formulations: one with quercetin and tiger nut extract, and another with luteolin, has a performance-enhancing effect in young men and women. A secondary aim was to test whether the combinations mangiferin-luteolin and mangiferin-quercetin protect skeletal muscle from the negative effects of ischemia/reperfusion applied immediately at the end of sprint exercise. We hypothesized that both mangiferin-containing supplements would enhance sprint performance.

## Materials and methods

### Subjects

Eighteen men and 17 women, all healthy and physically active agreed to participate in this investigation, but complete data was obtained from 17 men and 13 women (Table 1). After a familiarization and a pre-test phase lasting 4 weeks, the main experiment was performed to test the effects of two different combinations of polyphenolic supplement on performance during repeated sprint exercise. The study was carried out in accordance with the Declaration of Helsinki and was approved by the Ethical Committee of the University of Las Palmas de Gran Canaria (CEIH-2017-02). All subjects signed a written informed consent before entering the study. Subjects were requested to avoid strenuous exercise 48 h before the laboratory test and not to drink beverages containing caffeine or taurine during the 24 h preceding the test.

**Table 1:** Physical characteristics and ergoespirometric variables (mean ± *SD*).

	**Men (*n* = 17)**	**Women (13)**	***P***
Age (years)	22.7 ± 2.1	27.0 ± 2.2	0.005
Height (cm)	176.9 ± 4.2	164.4 ± 4.6	0.000
Weight (kg)	71.2 ± 5.2	56.5 ± 5.4	0.000
% body fat	18.4 ± 3.7	26.0 ± 4.9	0.000
Lean mass of both legs (kg)	19.8 ± 2.0	13.6 ± 2.5	0.000
Hemoglobin (g.dL^−1^)	15.0 ± 0.8	13.2 ± 0.9	0.000
HRmax (Beats/min)	191.7 ± 7.5	189.3 ± 0.7	0.567
VO_2_max (mL/kg/min)	47.5 ± 6.1	41.2 ± 6.1	0.005
VO_2_max (mL/kg LLM/min)	171.1 ± 16.3	170.4 ± 15.7	0.921
Wmax (W)	259.1 ± 32.7	177.7 ± 38.0	0.000
	**Constant-intensity test at 120% VO**_2_**max**		
Endurance time (s)	150.4 ± 40.1	132.0 ± 40.3	0.168
120% VO_2_max intensity (W)	303.8 ± 36.6	216.6 ± 40.5	0.000
Work (kj.kg^−1^ LLM)	2.30 ± 0.60	1.98 ± 0.60	0.086
O_2_ deficit (mL)	3362 ± 839	1880 ± 848	0.000
O_2_ deficit (mL.kg^−1^ BW)	47.2 ± 11.6	33.4 ± 11.4	0.001
O_2_ deficit/LLM	169.3 ± 35.9	137.9 ± 34.1	0.011
% Anaerobic Energy	33.6 ± 6.3	32.1 ± 5.8	0.527
	**30 s Wingate test**		
Wpeak_i_	1087.1 ± 86.5	753.0 ± 93.4	0.000
Wpeak_i_/kg	15.3 ± 1.2	13.4 ± 1.2	0.000
Wpeak_i_/LLM	55.4 ± 6.0	55.3 ± 6.5	0.979
Wmean	628.0 ± 65.6	417.3 ± 77.0	0.000
Wmean/kg	8.8 ± 0.8	7.4 ± 0.9	0.000
Wmean/kg LLM	31.9 ± 3.1	30.7 ± 3.0	0.270

*Wmax, maximal intensity during the incremental exercise test to exhaustion; Wpeak_i_, instantaneous peak power output during the Wingate test; LLM, lean mass of the lower extremities; Wmean, mean power output during a 30 s Wingate test; Accumulated VO_2_, total amount of O_2_ consumed); % Anaerobic Energy, percentage of the energy obtained through the anaerobic pathways*.

### Pre-tests

Body composition was determined by dual-energy x-ray absorptiometry (Lunar iDXA, GE Healthcare, Wisconsin; USA) as described elsewhere (Calbet et al., 1998). Subjects performed two familiarizations visits during which incremental exercise to exhaustion and a 30 s all-out sprint were performed. After familiarization, subjects reported to the laboratory to complete different tests on separate days. First, their peak VO_2_ (VO_2_peak), maximal heart rate (HRmax) and maximal power output (Wmax) were determined in normoxia (F_I_O_2_: 0.21, P_I_O_2_: 143 mmHg) with an incremental exercise test to exhaustion with verification (Poole and Jones, 2017). The test started with 3 min at 20 W, followed by 15 and 20 W increases every 3 min in women and men, respectively, until the respiratory exchange ratio (RER) was >1.0. After completion of the intensity with an RER ≤ 1.0, the intensity was increased by 10 and 15 W/min increase (women and men, respectively) until exhaustion. The intensity attained at exhaustion was taken at the maximal power output of the incremental exercise test (Wmax). At exhaustion, the ergometer was unloaded and subjects remained seated on the cycle ergometer pedaling at a slow speed (30–40 rpm) for 3 min. Thereafter, the verification test started at Wmax + 5 W for 1 min, followed by 4 and 5 W increase (women and men, respectively) every 20 s until exhaustion. Between 1 and 2 weeks later, subjects reported to the laboratory on two occasions separated by at least 1 week, to carry out a constant-intensity supramaximal exercise to exhaustion at 120% of VO_2_max. This test was used to determine the anaerobic capacity, as previously described (Morales-Alamo et al., 2015). The constant-intensity supramaximal exercise test with longer endurance time to exhaustion was retained as representative for each subject.

### Power output, oxygen uptake, and supramaximal exercise O_2_ demand and deficit

Power output during the sprint was reported as instantaneous peak power output (Wpeak) and mean power output (Wmean) throughout the duration of the sprints. Oxygen uptake was measured with a metabolic cart (Vyntus CPX, Jaeger-Carefusion, Hoechberg, Germany), calibrated according with high-grade certified gases provided by the manufacturer. Respiratory variables were analyzed breath-by-breath and averaged every 20 s during the incremental exercise tests (Calbet et al., 1997) and during the repeated sprints. The highest 20 s averaged VO_2_ recorded during the incremental test (i.e., including the verification phase) was taken as VO_2_peak.

The O_2_ demand during the sprints was calculated from the linear relationship between the last 20 s averaged VO_2_ of each load, from 80 W up to 80–90% of VO_2_max, while subjects were pedaling at 80 rpm. The accumulated oxygen deficit (AOD), representing the difference between O_2_ demand and VO_2_, was determined as previously reported (Calbet et al., 1997; Dorado et al., 2004).

### Main experiment

The volunteers were randomly assigned to three treatments, following a double-blind design, using a computer program. Treatment A, was a placebo condition (500 mg of maltodextrin per day); treatment B consisted in 140 mg of MLE (60% mangiferin) and 50 mg of luteolin per day; and treatment C contained 140 mg of MLE (60% mangiferin), 600 mg of quercetin and 350 mg of tiger nut extract per day (Table 2). The three treatments were divided in three daily doses administered every 8 h in methylcellulose capsules of identical appearance. The dose of quercetin was based on previous studies (Davis et al., 2010; Myburgh, 2014). The dose of mangiferin was based on a pharmacokinetic study showing oral absorption and mean residence time close to 7 h, after the ingestion of 0.1 g of pure mangiferin (Hou et al., 2012). The dose of luteolin was based on pharmacokinetic data obtained in humans after the administration of artichoke leaf extracts which are rich in luteolin (Wittemer et al., 2005) and the response observed after the administration of 100 mg of encapsulated luteolin in children (Tsilioni et al., 2015). Pilot studies showed no side effects from the administration of Zynamite in twelve volunteers, which had normal kidney, liver, hematological and biochemical variables in blood after two weeks of supplementation.

**Table 2:** Chemical composition of the plant extracts used in the supplements.

	***Mangifera indica L*. extract**	***Arachis hypogaea* extract**	***Sophora japonica* extract**	***Cyperus esculentus* extract**
Part of the plant used	Leaves	Shell	Bulbs, skin	Dry tubers
Bioactive compounds (%, w/w)	Mangiferin (≥60%)	Luteolin (≥90%)	Quercetin (≥90%)	HAF^a^ (≥5%)
	Homomangiferin (≤2.5%)			Oleic acid glyceryl ester^b^ (2:1) (≥91%)
	Isomangiferin (trace levels)			Linoleic acid glyceryl ester^b^ (≥7%)
	Sugars (≤10%)			Stigmaesterol^b^ (≥0.2%)
				Myricetin^b^ (≥0.2%)
				Sucrose (≤30%)
Moisture content (%, w/w)	≤ 7%	≤ 7%	≤ 7%	≤ 7%
Botanical or native ingredient (%, w/w)	*Mangifera i*. extract (100%)	*Arachis h*. extract (100%)	*Sophora j*. extract (100%)	*Cyperus e*. Extract (≥50%)
Non-botanical ingredient (%, w/w)	None	None	None	Potato maltodextrin (≤25%)
				Arabic gum (≤25%)

a *High Activity Fraction (HAF): Fraction soluble in ethyl acetate*.

b *Relative to the amount of the HAF*.

**Figure 1:**
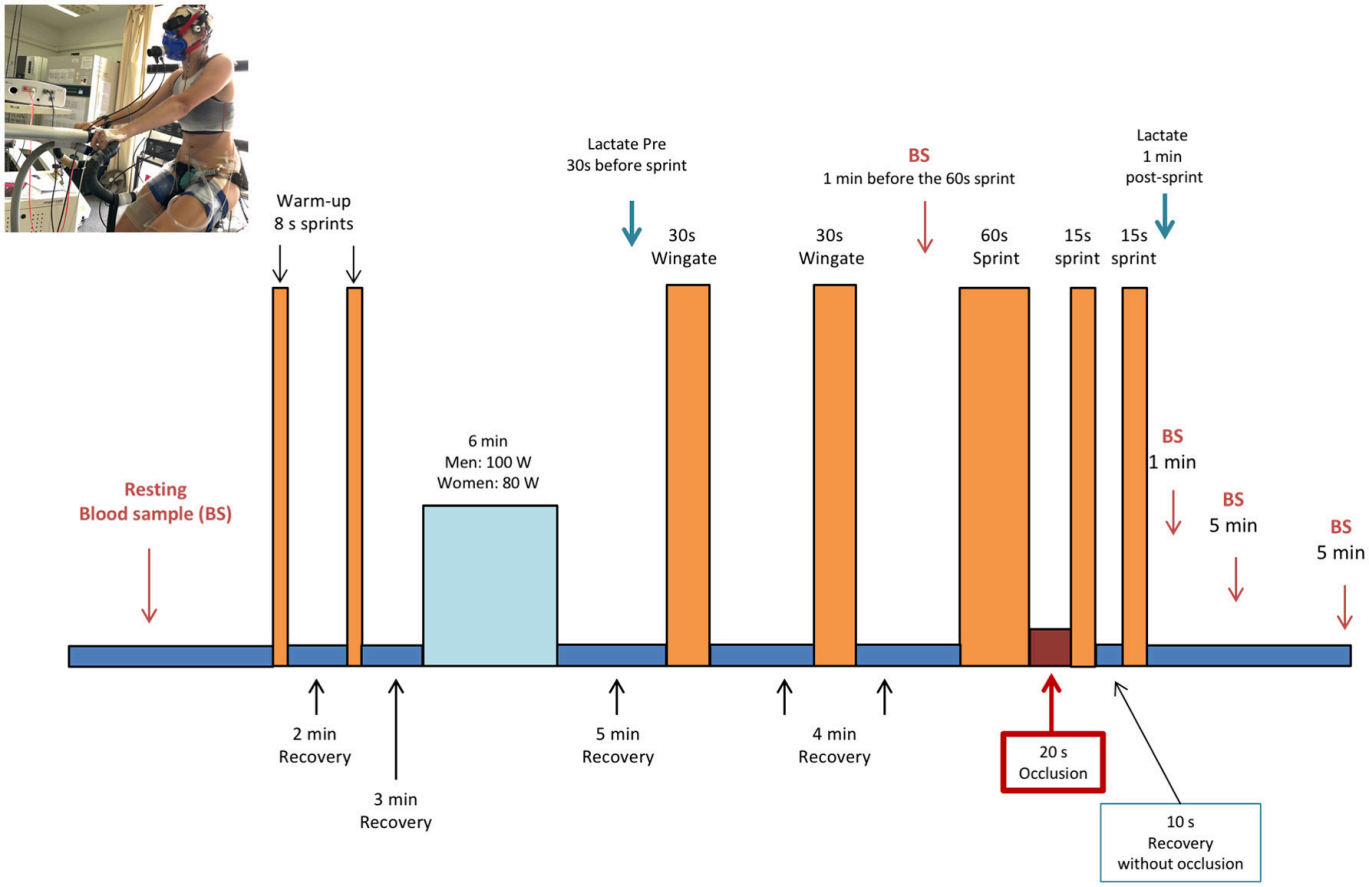
Experimental protocol (see text for details). Written consent was obtained from the volunteer that appears in the picture for the publication of the image.

Subjects started supplement intake 48 h before the main experimental days. On the day of experiment, subjects reported to the laboratory after a 10 h overnight fast, and 60 min before the start of the experiment ingested an additional dose of the supplement (i.e., 1/3 of the daily dose). During the following 60 min subjects were instrumented and a hand vein was catheterized and heated to obtain 10 ml of arterialized blood. Next, subjects were seated on the cycle ergometer and performed two warming-up 8 s sprints in isokinetic mode at 80 rpm, separated by a 2 min interval during which they pedaled with the cycle ergometer unloaded. After a 3 min period of unloaded pedaling, the load was increased to 80 W in women, and 100 W in men for 6 min (80 rpm, ergometer set in rpm-independent mode). This was followed by unloaded pedaling for 4.5 min. Then, subjects stopped pedaling and the ergometer was switched to isokinetic mode. At the 5th minute they performed a Wingate test (30 s all-out sprint in isokinetic mode at 80 rpm). This was followed by another 3.5 min of unloaded pedaling and another 30 s period, during which they stopped pedaling and the ergometer was switched to the isokinetic mode. At the 4th minute, a second 30 s Wingate test was performed, which was also followed by another 3.5 min of unloaded pedaling and another 30 s period of rest. Four min after the end of the second 30 s Wingate test, an all-out 60 s long sprint was carried out. At the end of the 60 s sprint, the circulation of both lower extremities was instantaneously occluded for 20 s by inflating bilateral cuffs at 300 mmHg as previously reported (Morales-Alamo et al., 2015; Torres-Peralta et al., 2016b; Figure 1). For this purpose, cuffs were placed around the thighs during the preparation phase, as close as possible to the inguinal crease, and were connected to a rapid cuff inflator before they seated on the cycle ergometer (SCD10, Hokanson E20 AG101, Bellevue, USA).

Ten seconds after the start of the occlusion a reverse countdown was given and the subjects prompted to start pedaling again as fast and hard as possible, with the ergometer in isokinetic mode for 15 s. At the start of the sprint the cuff was deflated to allow full reestablishment of the circulation during the subsequent exercise. At the end of the 15 s sprint, they pedaled slowly for another 5 s and then, stopped for 5 s to get ready for the final 15 s sprint. During the 10 s of recovery that followed the 15 s post-ischemia sprint, as well as during the 15 s final sprint, the circulation was open. A capillary blood sample was drawn from the ear lobe, previously hyperhemized with Finalgon® cream, to measure the concentration of lactate (lactate-Pro 2, Arkray, Valencia, Spain) 1 min after the last sprint.

Blood samples for hemoglobin concentration, blood gases, electrolytes and acid-base balance assessment were obtained from the heated hand vein at rest, 3 min after the second 30 s Wingate test, 1 min after the last sprint, and 5 and 30 min into the recovery period (ABL90, Radiometer, Copenhagen, Denmark).

#### Cerebral oxygenation

Cerebral oxygenation was assessed at rest and during exercise using near-infrared spectroscopy (NIRS, NIRO-200, Hamamatsu, Japan) employing spatial resolved spectroscopy to obtain the tissue oxygenation index (TOI) using a path-length factor of 5.92 (van der Zee et al., 1992). The NIRS optodes were placed on the right frontoparietal region at 3 cm from the midline and 2–3 cm above the supraorbital crest, to avoid the sagittal and frontal sinus areas (Curtelin et al., 2018). Using this optode placement the tissue oxygenation of the superficial frontal cerebral cortex is recorded. An additional optode was placed in the lateral aspect of the thigh at middle length between the patella and the anterosuperior iliac crest, over the middle portion of the *musculus Vastus Lateralis*. The rate of muscle deoxygenation upon occlusion was calculated by determining the maximal slope of the linear decay of TOI overtime. For this purpose, data were averaged every second and the slope TOI/time was calculated from the start of the occlusion to the end of occlusion, with a minimum interval of 4 s and a maximum of 20 s. Since the best linear fit was obtained with a 4 s interval, this was applied to all the occlusions.

#### Middle cerebral artery blood velocity

The mean blood velocity in the middle cerebral artery (MCAv_mean_), insonated through the trans-temporal window as described elsewhere (Rasmussen et al., 2007; Willie et al., 2011; Curtelin et al., 2018), was determined as an estimate of cerebral blood flow. Two Doppler 2 MHz transducers were applied bilaterally over the middle transtemporal window (Naqvi et al., 2013; Multi Box, DWL, Singen, Germany). Since both Doppler probes yield similar readings these were averaged for further analysis to reduce variability. In some subjects one MCA was insonated due to a mechanical failure of the second probe. A head harness was used to minimize potential movement artifacts. Resting cerebral oxygenation and MCAv_mean_ was calculated as the average of a 2 min collection period, while during exercise 5 s averages were generated and the average for the whole sprint reported. The NIRS and Doppler data were collected using a 16-channel data acquisition system (Power Lab ML880, ADInstruments), sampled at 200 Hz, and stored on a computer for subsequent analysis.

#### Power output

All pre-tests were performed on the same cycle ergometer (Lode, Corival, Groningen, The Netherlands), which maintains the exercise intensity constant despite variations in pedaling rate. During all tests subjects were requested to maintain a pedaling rate close to 80 rpm. For the main experiments, an isokinetic ergometer (Excalibur Sport 925900, Lode, Groningen, The Netherlands) was used. This ergometer was operated in a rpm-independent constant load during the warm-up and recovery phases and switched to isokinetic mode during the sprints, with the speed set at 80 rpm. During the isokinetic sprints, the subjects pedaled as fast and hard as possible, exerting as much force on the pedals as they could at each pedal stroke from the start to the end of the sprint, along which subjects were provided with strong verbal support. The servo-control brake system of the cycle ergometer adjusts continuously and almost instantaneously the braking force so the pedaling rate stays at 80 rpm during the whole sprint. In all instances, exhaustion was defined by the incapacity of the subject to maintain a pedaling rate above 50 rpm during 5 s, despite strong verbal encouragement or by a sudden stop in pedaling.

#### Oxygen demand and deficit

The O_2_ demand during the supramaximal exercise bouts was estimated from the linear relationship between the last min averaged VO_2_ of each load, from 20 to 40 W to the highest intensity with an RER < 1.00 in the incremental exercise test. The accumulated oxygen deficit (AOD), representing the difference between O_2_ demand and VO_2_, was determined as previously reported (Calbet et al., 1997; Dorado et al., 2004).

#### Assessment of pain and effectiveness of concealment

Subjects were requested to rate the level of pain felt during the occlusion from 0 to 10, being 10 the highest muscle pain ever suffered during or after exercise in their life. Likewise, at the end of the experiment subjects were asked about the kind of supplement they suspected they had received to check on the effectiveness of concealment. After placebo administration, 7 out of 30 subjects guessed correctly that they had placebo. Following B supplementation, 11 subjects out of 30, guessed correctly that they had polyphenols, and after supplement C, 16 out of 30 guessed correctly that they had polyphenols. Subjects were aware that polyphenols were present in two occasions and that in one occasion there would be placebo. They also knew that it was unknown whether polyphenols may improve or not performance. Notwithstanding, they generally reported to believe that they had taken polyphenols when they felt better during the whole experiment.

### Statistics

A sample size between 20 and 28 participants was required to provide adequate power to detect an improvement between 5 and 6% in peak power output (α = 0.05, β = 0.80; G^*^Power v.3.1.9.2). To allow for potential dropouts, we decided to recruit 36 volunteers. Variables were checked for normal distribution by using the Shapiro–Wilks test. A two-way repeated-measures ANOVA was used with two within-subjects factors: exercise bout (with five levels) and occlusion (with two levels: free or occluded recovery), and with sex as between-subjects factor. The Mauchly's test of sphericity was run before the ANOVA, and in the case of violation of the sphericity assumption, the degrees of freedom were adjusted according to the Huynh and Feldt test. When a significant main effect or interaction was observed, specific pairwise comparisons were carried out with the least significant difference post-hoc test. The relationship between variables was determined using linear regression analysis. Values are reported as the mean ± standard deviation of the mean (unless otherwise stated). *P* ≤ 0.05 was considered significant. Statistical analysis was performed using SPSS v.15.0 for Windows (SPSS Inc., Chicago, IL).

## Results

Men and women had comparable levels of fitness. Although men had a 15% greater VO_2_max per kg of body mass, the between-sex difference disappeared when the VO_2_max was expressed per kg of lean mass of the lower extremities. Likewise, men had 41% greater anaerobic capacity per kg of body mass, but this difference was reduced to 23% when expressed in relation to the lean mass of the lower extremities. Nevertheless, no significant between-sex differences were observed in the Wingate test when the values were normalized to the lean mass of the lower extremities.

### Effects on performance

**Figure 2:**
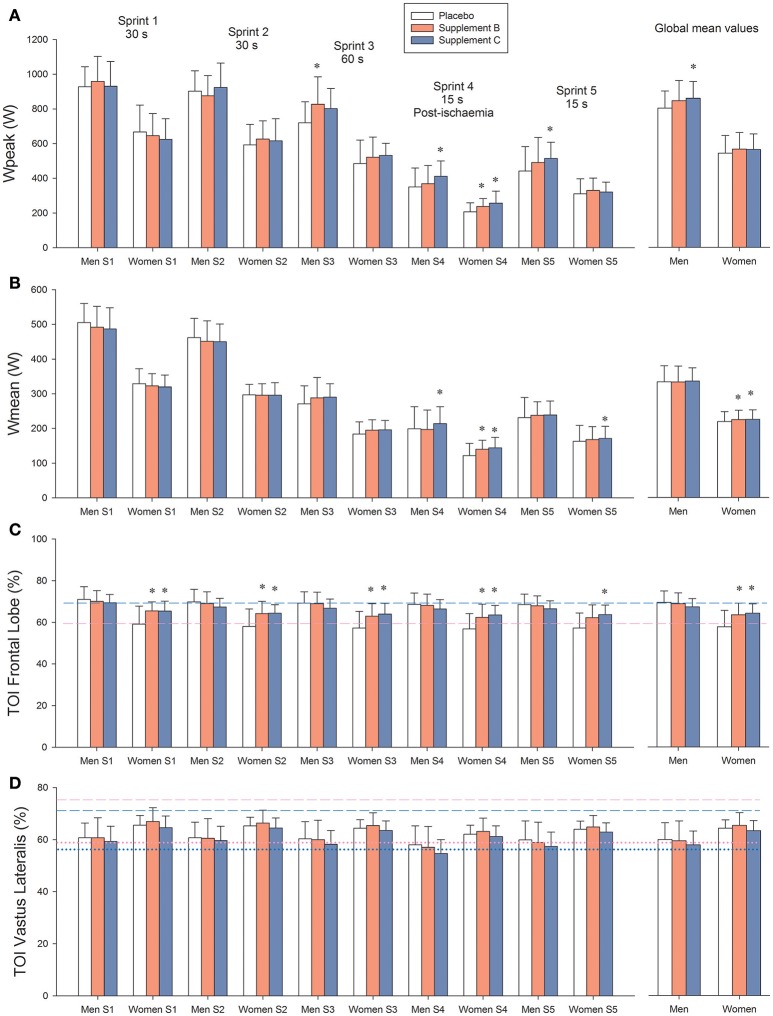
**(A)** Peak power output (Wpeak). **(B)** Mean power output (Wmean). **(C)** Brain oxygenation (Frontal lobe tissue oxygenation index: TOI). **(D)** *Vastus Lateralis* oxygenation index. Treatment A: placebo (500 mg of maltodextrin per day); treatment B consisted of 140 mg of mango leaves extract and 50 mg of luteolin per day; and treatment C contained 140 mg of mango leaves extract, 600 mg of quercetin, and 350 mg of tiger nut extract per day. Dashed lines in **(C**,**D)** represent the values recorded at rest. Dotted lines in **(D)** represent the values observed during the last 5 s of the occlusions after sprint 3, i.e., is the TOI value corresponding to “zero oxygenation.” Blue color: men, pink color: females. ^*^P < 0.05 compared with placebo.

Both supplements B and C enhanced performance during the latest sprints, i.e., those carried out with greater level of fatigue and lower functional reserve. Compared with placebo, when all sprints were averaged, supplements B and C increased Wpeak by 5.0 and 6.0%, respectively (Table 3 and Figure 2A). During the 60 s long sprint, supplements B and C increased Wpeak by 12.5 and 10.8%, respectively (*P* < 0.05). In the sprint performed after ischemia, supplement C increased Wpeak by 19.4% compared to placebo (*P* < 0.001) and by 10.2% compared to supplement B (*P* < 0.05).

**Table 3:** Ergoespriometric responses during repeated all-out sprints (mean, upper row, ± *SD*, lower row).

	**Sprint 1**			**Sprint 2**			**Sprint 3**			**Sprint 4**			**Sprint 5**			**ANOVA effects**				
	**W1A**	**W1B**	**W1C**	**W2A**	**W2B**	**W2C**	**W60A**	**W60B**	**W60C**	**W15A**	**W15B**	**W15C**	**W15FA**	**W15FB**	**W15FC**	**Sprint**	**Sprint × sex**	**Treat**	**Treat × sex**	**Sprint × Treat**
Wpeak (W)^b^	814.9	822.8	798.0	768.1	767.7	790.0	617.9	695.1^*^	684.4^*^	288.0	311.9	343.9^*^¶	385.3	421.0	430.4^*^	0.001	0.578	0.001	0.728	0.001
	185.8	207.7	202.1	194.2	167.2	204.4	172.6	207.4	167.0	113.3	106.1	111.2	135.0	142.0	125.3					
Wmean (W)^b^	428.5	419.0	414.3	390.4	383.7	383.0	233.4	247.8^*^	249.0^*^	165.4	172.5	183.9^*^¶	201.3	207.7	209.5	0.001	0.698	0.005	0.285	0.001
	101.3	98.8	98.3	94.5	92.1	89.6	62.4	66.9	58.5	65.6	53.2	53.8	62.5	51.7	50.4					
HR (beat.min^−1^)	156.6	155.9	158.7	158.6	158.5	161.0	169.3	168.5	170.1	173.3	174.9	176.2	173.3	175.0	178.1	0.001	0.082	0.92	0.27	0.3
	13.6	15.5	18.4	15.0	16.6	15.5	13.5	14.5	11.4	15.9	14.2	9.9	15.7	16.2	10.1					
VO_2_ (mL/min)	1006.3	1011.8	1010.4	1065.7	1063.6	1057.9	2415.1	2426.2	2429.1	540.1	549.2	550.7	635.7	641.9	644.4	0.001	0.001	0.91	0.4	0.99
	227.6	247.4	213.3	254.4	255.9	236.4	578.8	604.6	567.0	160.9	161.7	131.9	185.5	181.5	193.0					
VCO_2_ (mL/min)	2012.5	2023.7	2020.8	2131.3	2127.3	2115.7	2415.1	2426.2	2429.1	2160.3	2197.0	2202.8	2542.8	2567.5	2577.5	0.001	0.18	0.88	0.4	0.98
	455.2	494.7	426.7	508.8	511.7	472.9	578.8	604.6	567.0	643.7	646.7	527.7	741.9	725.8	772.2					
O_2_ deficit (mL)	1611.5	1601.0	1605.5	1367.1	1378.4	1332.3	810.0	927.2	768.1	57.0	75.9	90.8^*^	63.5	70.2	76.2	0.001	0.001	0.37	0.62	0.105
	520.6	536.4	495.1	456.8	445.9	440.2	427.2	531.7	432.3	94.6	142.9	108.5	115.7	120.7	123.4					
RER	1.02	1.04	1.02	1.03	1.04	1.03	0.97	0.98	0.96	1.16	1.18	1.16	1.04	1.05	1.07	0.001	0.012	0.574	0.61	0.62
	0.11	0.11	0.11	0.08	0.07	0.09	0.07	0.07	0.05	0.09	0.08	0.07	0.08	0.06	0.12					
V_E_ (L/min)	78.2	79.2	76.9	98.9	99.6	98.0	112.2	113.2	111.6	118.8	121.9	117.1	121.1	122.5	123.3	0.001	0.027	0.84	0.67	0.98
	22.5	22.1	23.1	23.7	25.7	28.0	29.4	30.3	30.5	35.0	33.4	37.0	36.7	35.3	36.8					
P_ET_O_2_ (mmHg)	115.5	114.6	112.6	119.7	119.7	119.3	119.8	120.1	120.0	122.6	122.9	119.5	119.9	119.9	120.5	0.001	0.34	0.21	0.633	0.405
	5.3	4.6	11.9	3.2	3.1	4.7	2.9	2.6	2.8	2.6	2.4	17.1	2.4	2.5	2.7					
P_ET_CO_2_ (mmHg)	29.0	30.5	29.9	25.6	25.9	26.3	24.2	24.1	24.4	24.6	24.7	24.3	25.7	25.7	25.7	0.001	0.099	0.6	0.6	0.34
	3.7	3.7	4.3	2.3	2.5	3.5	2.5	2.2	2.5	2.6	2.2	4.5	2.5	2.5	2.7					

Wpeak, peak power output; Wmean, mean power output; HR, heart rate; VO_2_, oxygen uptake; VCO_2_, CO_2_ production; RER, respiratory exchange ratio; V_E_, pulmonary ventilation; P_ET_O_2_, end-tidal O_2_ pressure; P_ET_CO_2_, end-tidal CO_2_ pressure; W1, first Wingate (30 s sprint); W2, second Wingate (30 s sprint); W60, 60 s sprint; W15, 15 s sprint post-ischemia; W15F, final 15 s sprint; Treat, treatment effect. A, Placebo; B, luteolin + Mangiferin; C, Mangiferin + Quercetin + Tiger nut extract (^b^statistical analysis done with logarithmically transformed data);

* P < 0.05 compared with placebo;

¶ *P < 0.05 compared with treatment B*.

When all sprints were combined, supplements B and C enhanced Wmean by 6.2 and 6.7%, respectively, compared with placebo (*P* < 0.01; Table 3 and Figure 2B). Consequently, the total amount of work performed was 2.4% higher following the ingestion of supplements B and C, compared with placebo in women (34.1 ± 4.3, 34.9 ± 4.1, and 34.9 ± 4.0 kJ, for placebo and supplements B and C, respectively, *P* < 0.05). The corresponding values in men were 51.7 ± 6.7, 52.1 ± 7.3, and 52.3 ± 5.8 kJ, respectively (*P* > 0.3). During the sprint performed after ischemia, supplement C enhanced Wmean by 11.2 (*P* < 0.001) compared with the placebo trial and 6.7% compared with supplement B (*P* = 0.012; Table 3).

### Pulmonary gas exchange

During the sprint after ischemia the level of pulmonary ventilation (V_E_) was higher than during the preceding (3rd sprint) and the subsequent sprint (5th sprint) (*P* < 0.01, for both comparisons). The increased V_E_ during the sprint after ischemia was associated with a higher end-tidal O_2_ pressure (P_ET_O_2_) compared with the 3rd and 5th sprints, and slightly lower end-tidal CO_2_ pressure (P_ET_CO_2_) in the 5th than 4th sprint during the placebo and B treatment conditions (all *P* < 0.01). After the administration of supplement C, ischemia did not alter V_E_, P_ET_O_2_, nor P_ET_CO_2_ during the 4th sprint, compared to the 3th and 5th sprints.

In women, the peak VO_2_ reached during the repeated sprints was 5.8% greater after the administration of supplements (mean of both trials) compared with the placebo trial (2,189 ± 334 and 2,316 ± 403 mL.min^−1^, for placebo and supplements, respectively, *P* = 0.012). No such an effect was observed in men (3,265 ± 406 and 3,318 ± 422 mL.min^−1^, placebo and supplements, respectively, *P* = 0.42).

Neither the accumulated VO_2_ nor the O_2_ deficit observed during the sprints were significantly altered by any of the treatments, when all sprints were analyzed conjointly. Nevertheless, during the 15 s sprint performed after ischemia, the *m. Vastus Lateralis* oxygenation index tended to be a slightly lower value after the administration of supplement C compared with placebo (*P* = 0.056).

There was a trend for an interaction between sprint and treatment for O_2_ deficit (*P* = 0.10). In fact, when the analysis was circumscribed to the sprints performed immediately following ischemia, the O_2_ deficit incurred was 2.7-fold greater after the ingestion of supplement C than after placebo in men (*P* = 0.001), while it remained at the same level in women. Pulmonary ventilation and gas exchange were similar in the three conditions (Table 3).

### Brain oxygenation

Resting brain oxygenation was lower in women than in men (*P* < 0.001). This was associated with lower P_ET_CO_2_ in women than in men (30.7 ± 2.6 and 34.2 ± 2.1 mmHg, in women and men, respectively, *P* < 0.001). In women, both supplements increased frontal lobe oxygenation at rest (59.4 ± 5.7, 64.9 ±3 .8, and 64.9 ± 6.4%, for placebo, supplement B and C, respectively, *P* < 0.05; for the comparisons of supplement B and C against placebo, treatment x sex interaction, *P* = 0.013). In men, brain oxygenation remained unchanged (69.3 ± 5.4, 69.1 ± 4.2, and 68.0 ± 4.4%, for placebo, supplement B and C, respectively, *P* > 0.50, for the comparisons of supplement B and C against placebo).

Brain oxygenation during the sprints was similar to that observed at rest. In women, brain oxygenation during the sprints was greater after the ingestion of supplements B and C than placebo (Figure 2). Likewise, during the 20 s ischemic recovery that followed the 60 s long sprint (sprint 3), brain oxygenation was higher after the ingestion of supplements B and C in women than in men (57.7 ± 7.2, 63.1 ± 6.0, and 64.0 ± 4.8%, for placebo, and supplements B and C, respectively, *P* < 0.05; for the comparison of supplement B and C with placebo: treatment x sex interaction *P* = 0.005). The corresponding values in men were not altered by the ingestion of supplements (68.0 ± 3.8, 67.9 ± 5.7, and 66.3 ± 4.3%, for placebo, and supplements B and C, respectively, *P* > 0.30).

### Muscle O_2_ extraction

During the occlusion, *Vastus Lateralis* TOI tended to be lower after the ingestion of supplement C compared to placebo (*P* = 0.082). The rate of muscle deoxygenation upon occlusion was similar for the three trials (1.18 ± 0.87, 1.21 ± 0.82, and 1.27 ± 0.71 TOI units.s^−1^, after the ingestion of placebo, supplement B and supplement C, respectively, *P* > 0.64). The level of TOI corresponding to “zero oxygenation” in the *m. Vastus Lateralis* was attained in about 5 s (5.0 ± 1.6, 5.0 ± 1.1, and 5.5 ± 1.8 s, for placebo and supplement B and C, respectively, *P* = 0.24; for the comparison of supplement C with placebo).

### Blood lactate, acid base-balance, and electrolytes

No significant differences in capillary blood lactate concentration 1 min after the last sprint were observed (14.1 ± 2.6, 13.9 ± 2.9, and 13.5 ± 3.2 mmol.L^−1^, after the ingestion of placebo, supplement B and supplement C, respectively). Exercise and recovery plasma Na^+^, Cl^−^, K^+^, Ca^2+^, glucose, and total bilirubin concentration was not modified by the supplements. Nevertheless, total bilirubin showed a trend for better recovery after the ingestion of supplement C compared to placebo (*P* = 0.06). None of the supplements modified the exercise-induced lactic acidosis in blood, which was similar for the three conditions (Table 4).

**Table 4:** Acid base balance, blood lactate, total bilirubin and electrolytes in arterialized blood (*n* = 10).

		**Resting**			**Three min after Wingate 2**			**One min after the last sprint**			**Five min into recovery**			**Thirty min into recovery**		
		**A**	**B**	**C**	**A**	**B**	**C**	**A**	**B**	**C**	**A**	**B**	**C**	**A**	**B**	**C**
Lac (mmol/L)	Mean	0.8	0.8	0.8	15.3	14.8	15.4	16.3	16.5	16.6	18.2	18.7	17.4	10.8	11.5	10.9
	*SD*	0.2	0.2	0.2	4.1	3.6	4.4	4.7	4.0	4.9	3.8	3.2	3.8	4.2	3.5	3.7
pH	Mean	7.411	7.416	7.414	7.190	7.200	7.190	7.143	7.127	7.145	7.130	7.119	7.127	7.291	7.277	7.285
	*SD*	0.043	0.029	0.031	0.058	0.046	0.058	0.056	0.067	0.075	0.076	0.072	0.077	0.076	0.063	0.065
pCO_2_ (mmHg)	Mean	41.0	41.3	37.5	35.7	34.1	33.3	42.2	45.5	37.9	27.0	28.8	30.0	30.4	30.8	31.0
	*SD*	4.1	3.5	12.9	8.0	7.0	5.9	9.9	13.9	8.7	2.4	4.5	6.4	4.2	4.1	4.7
SBC (mmol/L)	Mean	25.10	25.65	25.56	14.01	14.25	13.90	13.78	13.68	13.40	11.37	11.36	11.81	16.22	15.78	16.22
	*SD*	1.47	1.16	1.27	1.85	2.27	2.25	2.33	1.99	2.73	1.59	1.53	1.96	3.07	2.18	2.63
cBase (Ecf) (mmol/L)	Mean	1.34	1.99	1.74	−14.65	−14.61	−15.32	−14.46	−14.35	−15.69	−20.08	−19.91	−19.09	−11.59	−12.20	−11.65
	*SD*	1.56	1.64	1.65	2.99	4.09	4.05	4.27	4.01	5.07	3.00	2.99	3.90	5.02	3.79	4.48
Na^+^ (mmol/L)	Mean	140.5	140.4	127.3	145.5	144.8	144.6	147.8	148.1	147.0	144.2	144.5	143.4	140.9	141.1	140.1
	*SD*	1.2	1.1	42.2	3.7	1.7	1.9	3.1	2.4	1.7	2.5	1.5	1.7	1.1	1.4	1.1
Cl^−^ (mmol/L)	Mean	106.4	105.9	95.9	108.3	108.0	107.7	109.1	109.2	109.6	107.4	107.1	107.0	106.2	106.5	105.5
	*SD*	1.5	1.6	31.8	2.5	1.3	1.7	2.7	2.1	1.7	2.4	1.1	1.8	2.2	1.3	1.6
K+ (mmol/L)	Mean	3.9	3.9	3.6	3.9	3.8	3.8	4.7	4.8	5.1	3.5	3.8	4.0	3.8	3.8	3.8
	*SD*	0.3	0.2	1.2	0.6	0.5	0.4	0.4	0.5	0.9	0.2	0.6	1.1	0.2	0.2	0.2
Ca^2++^ (mmol/L)	Mean	1.21	1.21	1.11	1.26	1.24	1.25	1.30	1.30	1.29	1.26	1.25	1.25	1.21	1.21	1.21
	*SD*	0.04	0.03	0.37	0.03	0.04	0.03	0.04	0.05	0.05	0.04	0.04	0.04	0.03	0.04	0.03
tBil (μmol/L)	Mean	6.18	4.82	4.80	13.09	13.09	13.33	17.55	15.09	15.88	18.20	14.30	16.00	10.73	8.36	8.10^a^
	*SD*	7.51	9.34	7.11	8.68	9.14	8.65	9.06	9.90	9.64	9.99	4.64	6.65	7.63	9.99	7.06

*Lac, lactate; SBC, standard bicarbonate; cBase (Ecf), Base excess; tBil, Total bilirubin*;

a *P = 0.06 compared with placebo*.

### Pain during post-sprint ischemia

When both conditions with polyphenols were averaged, the level of pain reported was lower compared with the placebo condition (7.1 ± 1.8 and 6.7 ± 2.0 arbitrary units, for the placebo and the mean of the B and C conditions, respectively, *P* = 0.068).

## Discussion

This study shows that the ingestion of two supplements containing a mango leaf extract rich in mangiferin enhances performance in humans during high intensity exercise. Moreover, the combined MLE/quercetin/tiger nut extract had a remarkable effect increasing peak power output after ischemia/reperfusion, with a similar effect in men and women. In women, MLE-containing supplements improved brain oxygenation at rest and during exercise, and increased peak VO_2_ during high-intensity exercise.

Although, the main mechanism eliciting the ergogenic effect of the two MLE-containing supplements remains to be determined, our findings provide indirect evidence for an enhancement of performance without additional consumption of oxygen, suggesting either better muscle energy efficiency and/or enhanced capacity to recruit the exhausted muscle fibers by the central nervous system, with increased production of energy through the anaerobic pathways. Moreover, in agreement with our hypothesis, a trend for better muscular extraction of O_2_ was also observed in the sprints performed after ischemia/reperfusion when the subjects had taken the combined MLE/quercetin/tiger nut extract. In agreement with previous findings, ischemia elicited a slightly higher V_E_ response to sprint, which is consistent with an increased stimulation of the metaboreflex as previously explained (Morales-Alamo et al., 2015; Torres-Peralta et al., 2016b), and as shown by experiments using fentanyl blockade of III/IV muscle afferents (Dempsey et al., 2014). The combined MLE/quercetin/tiger nut extract suppressed this additional hyperpneic response observed during the sprints after ischemia. Interestingly, high interstitial accumulation of K^+^ and H^+^ may elicit pain via stimulation of III/IV muscle afferents (Kniffki et al., 1978; Mense, 1996). Here, the MLE-containing extract supplement tended to reduce the pain evoked by the occlusions. The attenuation of metaboreflex responses by MLE/quercetin/tiger nut extract is an important effect, since exaggerated responsiveness of III/IV have been shown to limit exercise performance in patients with heart failure (Ives et al., 2016; Keller-Ross et al., 2016) and chronic obstructive pulmonary disease (Gagnon et al., 2012).

### Mango leaves extract rich in mangiferin enhances performance during repeated prolonged sprints

The two supplements containing MLE had positive effects on performance, however, our data point toward some superiority of the MLE/quercetin/tiger nut extract over the combination MLE/Luteolin, particularly regarding the effects on ischemia/reperfusion. Although luteolin attenuates the ischemia/reperfusion injury in several tissues (Karakaş et al., 2014; Hong et al., 2017; Liu et al., 2017; Du et al., 2018) it remains unknown whether luteolin prevents the ischemia/reperfusion injury in skeletal muscle. In contrast, at least one study has shown that quercetin protects skeletal muscle from ischemia/reperfusion injury in rodents submitted to ischemia for 3 h (Ekinci Akdemir et al., 2016). Although the present experimental design does not allow partitioning the contribution of each polyphenol to the observed effects, the fact that an increase in performance was observed when MLE was present points toward mangiferin as the main compound responsible for the ergogenic effect. Moreover, quercetin supplementation during 1 week in 12 × 30 m running sprints has been reported to reduce performance (Abbey and Rankin, 2011).

### Potential mechanisms accounting for the performance-enhancing effects of MLE formulations

It has been shown in cell cultures that mangiferin activates pyruvate dehydrogenase (PDH) resulting in reduced lactate production and increase carbohydrate oxidation (Apontes et al., 2014). In contrast, no changes in exercise blood lactate responses nor substrate oxidation (data not shown) were observed in the present investigation during submaximal exercise. Although the energy efficiency during submaximal exercise was not significantly altered (data not shown), we cannot rule out an improvement of the contractile efficiency during repeated high intensity exercise after the administration of MLE. Muscle energy efficiency is reduced during high intensity exercise by several mechanisms which include, among others, increased recruitment of less efficient type II muscle fibers, lactic acidosis, electrolyte alterations, and RONS (Fitts, 1994; Westerblad and Allen, 2011; Morales-Alamo and Calbet, 2014). During high intensity exercise RONS are produced due to both the high mitochondrial respiratory rate and the activation of the anaerobic metabolism (Morales-Alamo et al., 2013; Morales-Alamo and Calbet, 2014). RONS may contribute to muscle fatigue by two main mechanisms: by reducing calcium sensitivity and/or reducing calcium release from sarcoplasmic reticulum (Bruton et al., 2008). In cardiac myofilaments, xanthine oxidase reductase inhibition enhances myofilament Ca^2+^ sensitivity, which may result in greater force production if the required energy is available. A similar effect might have been produced by the MLE-containing supplements in the present investigation.

Excessive RONS production could reduce mitochondrial P/O ratio, while antioxidants may favorably influence mitochondrial function improving efficiency (Clerc et al., 2007). Moreover, the ingestion of antioxidants before sprint exercise reduces the level of protein carbonyls in muscle and plasma (Morales-Alamo et al., 2012) and lowers the glycolytic rate (Morales-Alamo et al., 2017) without a detrimental effect on performance (Morales-Alamo et al., 2012, 2017).

The three polyphenols tested here have free radical quenching capacity (González-Gallego et al., 2010; Braakhuis and Hopkins, 2015; Luo et al., 2017; Rauf et al., 2017), being also inhibitors of XO (Pinto et al., 2005; Paredes-Gonzalez et al., 2015; Niu et al., 2016) and NOX (Makino et al., 2013; Xia N. et al., 2014). However, no study in humans has shown that antioxidants, even when administered intravenously are capable of enhancing peak power output during repeated prolonged sprint exercise. Thus, although the antioxidant properties of the supplements tested here may have contributed to enhance performance, other mechanisms must be involved, since a wide variety of antioxidants have previously failed to enhance peak power output in humans and none have shown these properties in the fatigued state.

To boost performance in a fatigued muscle greater calcium release is needed to enhance the number of cross-bridges that can be established, but also a faster calcium reuptake is required to shorten the relaxation phase. *In vitro* caffeine can enhance force in fatigued muscle by boosting Ca^2+^ release, but the dose needed would be lethal for humans (Fredholm, 1995). In this regard, mangiferin, a major component of MLE, shares some common intracellular mechanisms of action with caffeine, which may facilitate calcium release in the fatigued state (i.e., when Ca^2+^ release is depressed). Like caffeine and beta-agonists, mangiferin may increase cAMP, and through the activation of protein kinase A (PKA), stimulate SERCA activity. At rest phospholamban (PLN) inhibits SERCA activity, but when phosphorylated by PKA or Ca^2+^/calmodulin-dependent protein kinase (CaMKII), PLN dissociates from SERCA, and SERCA activity increases (MacLennan and Kranias, 2003). However, at tolerable doses it is unlikely that caffeine can alter skeletal muscle metabolism in humans (Desbrow et al., 2009) and the main mechanism of the ergogenic action of caffeine is supposed to rely on its effect on the central nervous system, by enhancing muscle activation (Behrens et al., 2015) and decreasing the perception of effort (de Morree et al., 2014). Interestingly, in our experiment the mangiferin-containing supplements reduced the level of pain perceived during post-exercise ischemia. Whether this may positively influence the corticospinal drive during sprint exercise remains to be determined.

Although caffeine may enhance performance during prolonged exercise and team-sport activities its capacity to enhance power and strength is debated (Davis and Green, 2009; Goldstein et al., 2010). Moreover, there is no evidence supporting an ergogenic effect of caffeine during episodes of ischemia/reperfusion which may occur in some sport disciplines. Unlike caffeine, which may cause hypokalemia in athletes (Eichner, 2011), no effect of mangiferin on plasma potassium was observed here.

### Brain oxygenation and fatigue

Reduction in brain oxygenation has been associated with fatigue in several studies (Smith and Billaut, 2010; Torres-Peralta et al., 2016a; Santos-Concejero et al., 2017; Curtelin et al., 2018). Moreover, at exhaustion during exercise in hypoxia improving the oxygenation of the brain (and upper body) by swiftly raising the F_I_O_2_, while maintaining the lower extremities deoxygenated by instantaneously occluding the circulation, was associated with improved performance (Morales-Alamo et al., 2015), which supports a mechanistic link between brain oxygenation and fatigue during sprint exercise in a fatigued state (Torres-Peralta et al., 2016a). Here we show that MLE-containing supplements consumed before sprint exercise may counteract fatigue by improving brain oxygenation, at least in women. The reason why women were more sensitive to this effect should be addressed in new experiments, but may be related to phytoestrogen dependent protection of endothelial function, by an NO-dependent mechanism (Chen et al., 1999), similar to that of resveratrol (Xia N. et al., 2014), which has been shown to enhance brain perfusion in postmenopausal women (Klinge et al., 2005) and young adults (Wightman et al., 2015). In agreement with our results, it has been shown that resveratrol may improve frontal lobe oxygenation despite unchanged Doppler-measured middle cerebral artery velocity at rest in young adults (Wightman et al., 2015).

### The combination of mango leaf extract with quercetin has a strong protective effect of muscle functional capacity when exhausted skeletal muscles are submitted to ischemia/reperfusion

In the present investigation, we have tested for the first time in humans the potential protective effects of an empirical polyphenol combination (MLE + quercetin + tiger nut extract) on functional deterioration induced by ischemia/reperfusion. Estrogens (and phytoestrogens) may protect against ischemia/reperfusion injury through the activation of estrogen receptor (ERα) and the downstream signaling cascade phosphatidylinositol-3-OH kinase (PIK3)/Akt to promote cell survival and through PIK3/eNOS to stimulate endothelial NO release, the latter resulting in vascular protection (Chen et al., 1999). Both luteolin and mangiferin are known inhibitors of xanthine oxidoreductase (XOR), which is considered responsible for part of damage generated by ${\text{O}}_{2}^{-}$ during reperfusion, due to the formation of ·OH and ONOO^−^ radicals by XOR catalytic activity (Berry and Hare, 2004).

In summary, this study shows that the MLE 60% mangiferin (Zynamite) has a remarkable ergogenic effect increasing muscle power in fatigued subjects, without increasing the consumption of oxygen, submaximal exercise efficiency or submaximal and maximal blood lactate concentrations. This type of response is expected for a compound acting on the central nervous system. We have also shown for the first time in humans that MLE combined with quercetin and tiger nut extract assist in maintaining skeletal muscle function during ischemia/reperfusion, strongly suggesting that this combination is also acting directly on the skeletal muscles. Further studies should explore whether MLE/quercetin/tiger nut extract might have clinical application to prevent ischemia-reperfusion damage in patients during surgery or after post-embolism reperfusions.

## Disclosures

This study has been partly financed by Nektium Pharma, who kindly provided the phenolic compounds.

## Author contributions

JC, JW, and NG: conception and design of the experiments; DC, MG-R, MM-R, MP-V, VG-A, and DM-A: pre-testing, experimental preparation, and data collection; MG-R, MM-R, MP-V, VG-A, DM-A, LL-R, and JC: data analysis. The first draft of the manuscript was written by MG-R and JC. All co-authors edited and proofread the manuscript and approved the final version.

### Conflict of interest statement

Nektium Pharma, a nutraceutical company has supplied the polyphenols tested in this study and has financed partly the cost of the experiments. The execution of the experiments and interpretation of the results have been carried out using a double-blind design with complete freedom by the scientific team of the University of Las Palmas de Gran Canaria. The authors declare that the research was conducted in the absence of any commercial or financial relationships that could be construed as a potential conflict of interest.
